# Continuous immunosuppression is required for suppressing immune responses to xenografts in non-human primate brains

**DOI:** 10.1186/s13619-024-00191-0

**Published:** 2024-04-07

**Authors:** Su Feng, Ting Zhang, Zhengxiao He, Wenchang Zhang, Yingying Chen, Chunmei Yue, Naihe Jing

**Affiliations:** 1 Guangzhou National Laboratory, Guangzhou, 510005 China; 2 Shanghai General Hospital, Shanghai Jiao Tong University School of Medicine, Shanghai, 200080 China; 3 National Clinical Research Center for Eye Disease, Shanghai, 200080 China; 4 Shanghai Key Laboratory of Ocular Fundus Diseases, Shanghai, 200080 China; 5 Suzhou Yuanzhan Biotechs, Suzhou, 215000 China

**Keywords:** Cell transplantation, Xenograft, Immune response, Non-human primate brain, Human induced neural stem/progenitor cells (iNPCs)

## Abstract

Continuous immunosuppression has been widely used in xenografts into non-human primate brains. However, how immune responses change after transplantation in host brains under continuous immunosuppressive administration and whether immunosuppression can be withdrawn to mitigate side effects remain unclear. Human induced neural stem/progenitor cells (iNPCs) have shown long-term survival and efficient neuronal differentiation in primate brains. Here, we evaluate the immune responses in primate brains triggered by human grafts. The results show that the immune responses, including the evident activation of microglia and the strong infiltration of lymphocytes (both T- and B-cells), are caused by xenografts at 4 months post transplantation (p.t.), but significantly reduced at 8 months p.t. under continuous administration of immunosuppressant Cyclosporin A. However, early immunosuppressant withdrawal at 5 months p.t. results in severe immune responses at 10 months p.t. These results suggest that continuous long-term immunosuppression is required for suppressing immune responses to xenografts in primate brains.

**Supplementary Information:**

The online version contains supplementary material available at 10.1186/s13619-024-00191-0.

## Background

Cell-based therapies are promising interventions for neurodegenerative diseases, such as Alzheimer’s disease (AD) (Duncan and Valenzuela 2017; Yue et al. 2022), Parkinson’s disease (PD) (Barbuti et al. 2021; Barker et al. 2017; Cha et al. 2023) and amyotrophic lateral sclerosis (ALS) (Lin et al. 2022; Sironi et al. 2023), in which mass neurons are dysfunctional or lost at late stages. Human induced pluripotent stem (iPS) cells, which can be derived from a patient’s own cells at any age, hold the potential to be donor cells for patients with neurodegenerative disease. Patient-specific iPS cells can reduce the risk of immune rejection, facilitate long-term engraftment without the need for immunosuppression, and circumvent the ethical hindrances of using brain tissues from aborted fetuses (Madrid et al. 2021). However, even though patient-specific iPS cells have these advantages, they are unlikely to serve as donor cells in standard therapies because of the high cost and long preparation time of iPS cells for each patient (Turner et al. 2013). On the other hand, disease-specific and “off-the-shelf” donor iPS cells are more accessible for patients in need of cell transplantation. Prior to the clinical application of cell therapies for patients, the efficacy and safety of cell transplantation must be optimized in animal models. Compared with rodents, non-human primates are closer to humans in multiple aspects, such as similarities in genomics, neuroanatomy, neurophysiology, immunogenetics, and age-related changes in immune function (Grow et al. 2016; Sereno and Tootell 2005). Therefore, non-human primates hold the great potential as host animal models in preclinical trials of stem cell-based therapies.

Accompanying xenotransplantation, the inevitable problem is that the recipient's immune system will be activated to induce the immune rejection of grafted cells. Immunodeficient or humanized mice are usually used for human stem cell transplantation to prevent graft failure (Tanner et al. 2014; Zhang et al. 2019), but there are no available immunodeficient non-human primate models for evaluating the effectiveness of transplanted human cells. To prolong the survival of grafted cells and preserve cell function, chronic immunosuppressive maintenance is traditionally needed. However, long-term immunosuppressive treatment is accompanied by undesirable side effects, such as nephrotoxic effects, hypertension, and hyperlipidemia (Denton et al. 1999; Diehl et al. 2017; Mecadon 2021). Furthermore, chronic immunosuppression destroys the host immune system and increases opportunistic infections. Thus, a well-balanced immunosuppressive strategy is imperatively needed for graft survival and functioning.

Cyclosporin A (CsA) is a strong calcineurin inhibitor of the T-cell response that blocks interleukin 2 (IL-2) production (Hricik 2015) and is one of the mainstays for the prevention of graft rejection (Allison 2016; Azzi et al. 2013; Otsuka et al. 2021). In previous studies in which non-human primates served as hosts in cell transplantation, CsA was usually applied as an immunosuppressive drug for primates to modulate their immune system to avoid immune reactions (Emborg et al. 2013b; Gonzalez et al. 2015; Kriks et al. 2011). Although these studies indicate partially successful engraftment, the data are restricted to only 1–3 months after transplantation, and the immune responses are not well elucidated. In a recent study of parkinsonian marmosets transplanted with iPSC-derived dopaminergic neurons, functional engraftment was observed at 6 months after transplantation under the continuous immunosuppression of tacrolimus (also known as FK506) until necropsy (Daadi et al. 2024). It is still unknown how immune responses change over time after transplantation under long-term continuous administration of CsA. Moreover, whether a brief course of immunosuppressive administration, which helps reduce the side effects caused by CsA, is enough to reach the desired goal in avoiding graft rejection remains unclear.

In our previous study, we documented that transplanted human induced neural stem/progenitor cells (iNPCs) could successfully differentiate into mature and electrophysiologically functional neurons and survive long-term in primate brains under the administration of the immunosuppressant CsA (Feng et al. 2022). In this follow-up study, we further evaluated the immune response in the host primate brain with xenogenic cells at 4 months, 8 months, and 12 months post transplantation (p.t.) under the condition of continuous treatment with CsA or at 10 months with CsA treatment only within the first 5 months. We found that long-term immunosuppression mitigated the infiltration of microglia and lymphocytes into the human grafts, while early CsA withdrawal resulted in more severe immune responses. Therefore, the results revealed the necessity of long-term continuous immunosuppressive treatment to prevent graft rejection and mitigate immune responses in non-human primates transplanted with human iNPCs.

## Results

### The approach for the administration of cyclosporin a in cynomolgus monkeys transplanted with human iNPCs

**Fig. 1:**
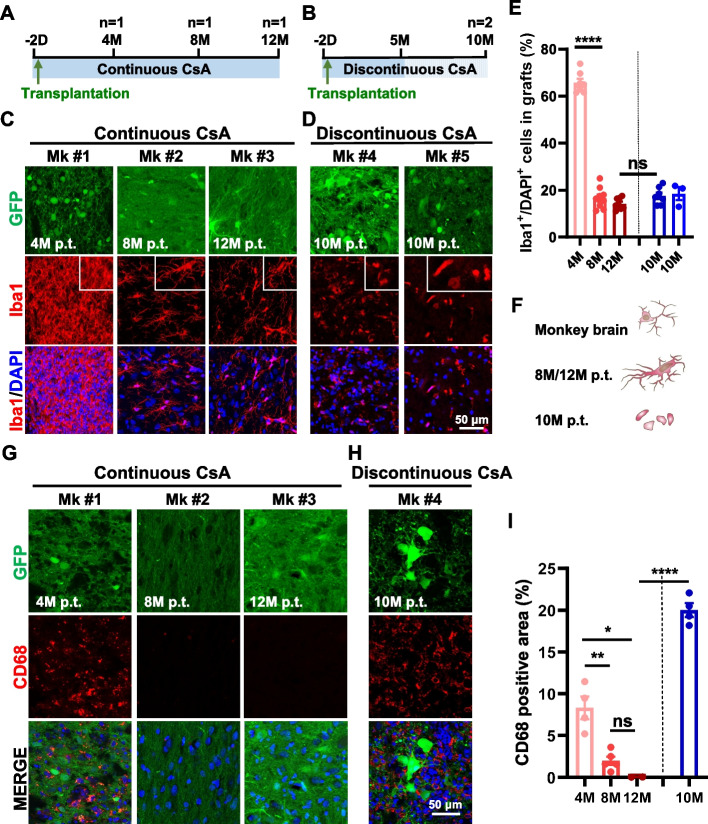
The activation and infiltration of microglia into human grafts. **A**, **B** Schematic diagram of human iNPCs transplantation, immunosuppressive strategy of Cyclosporin A, and time points for evaluation. **C**, **D** Immunofluorescent analysis of IBA1^+^ microglia in human grafts of Mk #1 at 4 months, Mk #2 at 8 months, and Mk #3 at 12 months p.t. with continuous CsA treatment, and Mk #4 and Mk #5 at 10 months p.t. with discontinuous CsA treatment. Cell nuclei were counterstained with DAPI. **E** Percentages of IBA1^+^ microglia in human grafts shown in (**C** and **D**). **F** The morphologies of homeostatic microglia in the monkey brain, activated microglia in human grafts at 8/12 months p.t., and phagocytic microglia at 10 months p.t. **G**, **H** Immunofluorescent analysis of CD68^+^ microglia/monocytes in human grafts of Mk #1 at 4 months, Mk #2 at 8 months, and Mk #3 at 12 months p.t. with continuous CsA treatment, and Mk #4 at 10 months p.t. with discontinuous CsA treatment. Cell nuclei were counterstained with DAPI. **I** Quantification of CD68^+^ area percentage per field shown in (**G**, **H**). Scale bars: 50 μm in (**C**, **D**) and (**G**, **H**). Data are represented as the mean ± SEM. *p < 0.05, **p < 0.01, ****p < 0.0001, ns (not significant)

Human induced neural stem/progenitor cells (iNPCs), generated from immobilized adult peripheral blood mononuclear cells (PB MNCs), showed strong survival capacity and neuronal differentiation property in non-human primate brains under continuous immunosuppressive treatment (Feng et al. 2022). However, it is not clear how immune responses change in the host brain caused by human iNPC transplantation with continuous treatment of the immunosuppressant Cyclosporin A (CsA). To address this question, we recruited three adult cynomolgus monkeys aged 10 to 12 years old for transplantation of GFP-labelled human iNPCs. All three monkeys received oral CsA delivery from 2 days before cell transplantation until euthanasia at 4 months, 8 months, and 12 months p.t. for monkey #1 (Mk #1), Mk #2 and Mk #3, respectively (Fig. 1A). Furthermore, another two monkeys (Mk #4 and Mk #5) were recruited to investigate whether a discontinuous immunosuppressive strategy has the same efficacy in suppressing immune responses during the process of human iNPCs differentiation into neurons. Therefore, CsA withdrawal was performed for Mk #4 and Mk #5 after 5 months and then they were euthanatized at 10 months p.t. (Fig. 1B).

It has been shown the differentiation of neurons and astrocytes derived from transplanted human iNPCs at all four different time points in our previous study (Feng et al. 2022). The mature neuronal marker neurofilament heavy chain (NF-H) remained to be detected in the grafts of Mk #4 and Mk #5 (Figure S1A), even the immunosuppression was withdrawn. Moreover, like the expression of SYNAPTOPHYSIN in the grafts under continuous treatment of CsA (Feng et al. 2022), SYNAPTOPHYSIN immunoreactivity was also observed in 10-month grafts under discontinuous treatment of CsA in present study (Figure S1B). Interestingly, under the continuous treatment of CsA, the estimated area of grafts in hemispheres became bigger from 4 to 12 months p.t., while the area of 10-month grafts from Mk #4 and Mk #5 under discontinuous immunosuppression was visibly smaller (Figure S2). Together, these data indicated that the differentiation and maturation of grafted cells were not affected by the immunosuppressant regiment, but massive grafted cells were lost after CsA withdrawal.

### Continuous CsA administration reduced the infiltration of microglia into the human grafts

Microglia, the brain-resident immune cells, work as key players in brain immunological responses (Davalos et al. 2005; Nimmerjahn et al. 2005). To investigate the immune response of xenotransplantation to the monkey brain, we first evaluated the microglia in the grafts by the general microglia marker IBA1. The immunofluorescent study showed that abundant microglia infiltrated into the human grafts at 4 months p.t., probably caused by acute physical damage and xenografts of human cells. The accumulation of microglia indicates the immune response to central nervous system insults (Gómez-Nicola et al. 2013; Graeber et al. 1998). After the acute inflammatory response, the infiltration of microglia into the grafts was significantly reduced at 8, 10 and 12 months p.t. (Fig. 1C, D and E), under either continuous or discontinuous CsA treatment. In addition to microglial accumulation, the morphologies of microglia reflect the different activation stages in response to disturbed homeostasis (Walker et al. 2014). In comparison to homeostatic microglia in the healthy monkey brain, ramified microglia in the grafts at 8 and 12 months p.t. had larger fusiform cell bodies and longer and bulkier branches (Fig. 1C and F), which showed their activated stage responding to the grafted human cells. Compared with continuous CsA treatment monkeys, microglial infiltration was not significantly increased in either Mk #4 or Mk #5, in which CsA administration was withdrawn at 5 months p.t. (Fig. 1C, D and E). However, the microglia were reactivated to phagocytic macrophages, losing their branches and only harboring microglial cell bodies (Fig. 1D and F), suggesting that these cells might phagocytize the grafted foreign cells and cause graft failure. These results revealed that continuous CsA administration could suppress the microglial immune response.

Next, we examined the expression of CD68 (for microglia/macrophages) in the human grafts. Similar to IBA1^+^ microglia, large numbers of CD68^+^ microglia/macrophages were also found in the grafts at 4 months p.t., but they were almost extinct at 8 and 12 months p.t. under continuous administration of CsA (Fig. 1G and I). Whereas CsA withdrawal at 5 months p.t. resulted in more abundant expression of CD68 in microglia/macrophages at 10 months p.t. (Fig. 1G, H and I). Additionally, the immunofluorescent study of HLA-DR (human leukocyte antigen-D related) showed a similar expression pattern with CD68—significantly higher expression of HLA-DR in the grafts at 4 and 10 months p.t. and extremely lower at both 8 and 12 months p.t. (Figure S1).

Together, the above data indicate that xenotransplantation stimulates the activation and accumulation of microglia in grafts. Continuous CsA treatment can suppress the microglial immune response, while CsA withdrawal at 5 months p.t. is not enough to maintain immunosuppression.

### Continuous CsA administration could suppress the infiltration of T and B lymphocytes into human grafts

**Fig. 2:**
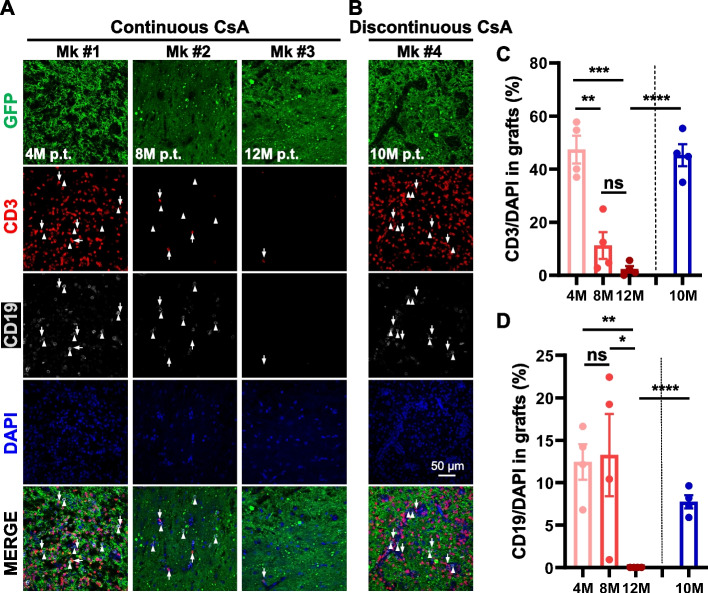
The infiltration of CD3^+^ T and CD19^+^ B lymphocytes into human grafts. **A**, **B** Immunofluorescent analysis of CD3^+^ T (arrow) and CD19^+^ B (arrowhead) lymphocytes in human grafts of Mk #1 at 4 months, Mk #2 at 8 months, and Mk #3 at 12 months p.t. with continuous CsA treatment, and Mk #4 at 10 months p.t. with discontinuous CsA treatment. Cell nuclei were counterstained with DAPI. **C**, **D** Percentages of CD3^+^ T and CD19^+^ B lymphocytes in human grafts of Mk #1, Mk #2, Mk #3, and Mk#4 shown in (**A** and **B**). Scale bars: 50 μm in (**A**, **B**). Data are represented as the mean ± SEM. *p < 0.05, **p < 0.01, ***p < 0.001, ****p < 0.0001, ns (not significant)

Activated microglia may release signals and trigger the recruitment of circulating immune cells (Kwon 2022). Therefore, we further detected the infiltration of lymphocytes into the grafts, including T and B lymphocytes. Double immunofluorescence staining of CD3 (for T lymphocytes) and CD19 (for B lymphocytes) was performed on monkey brain slides harboring GFP^+^ grafts. The results showed that approximately 47% of cells in the grafts were CD3^+^ T-cells at 4 months p.t. (Fig. 2A and C). Under the continuous administration of CsA, the ratio of infiltrated CD3^+^ T cells was significantly decreased to ~ 11% at 8 months p.t. and even to ~ 2% at 12 months p.t. (Fig. 2A and C). However, for Mk #4, CsA withdrawal at 5 months p.t., ~ 45% of cells were CD3^+^ T-cells in grafts at 10 months p.t., indicating an extreme increase in infiltration compared to that at 12 months p.t. (Fig. 2A, B and C). Moreover, approximately 13% of cells were CD19^+^ B-cells infiltrating into the grafts at 4 and 8 months p.t., but very few CD19^+^ B-cells could be detected at 12 months p.t. under continuous administration of CsA (Fig. 2A and D). Nevertheless, B-cell infiltration at 10 months p.t., under the discontinuous administration of CsA, was significantly higher than that at 12 months p.t. under continuous CsA treatment (Fig. 2A, B and D). Collectively, continuous CsA treatment showed its role in mitigating the infiltration of lymphocytes into human grafts in the host primate brain. In contrast, CsA treatment in the first five months did not suppress the infiltration of lymphocytes into the grafts.

### Continuous CsA administration could suppress the infiltration of CD4^+^ T-cells and CD8^+^ T-cells in human grafts

**Fig. 3:**
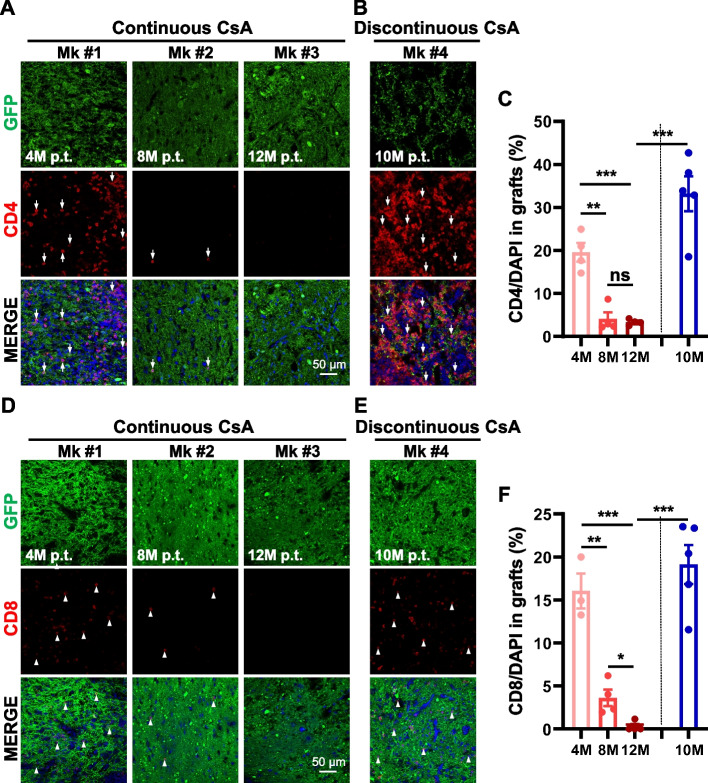
The infiltration of CD4^+^ helper and CD8^+^ cytotoxic T lymphocytes into human grafts. **A**, **B** Immunofluorescent analysis of CD4^+^ helper T (arrow) lymphocytes in human grafts of Mk #1 at 4 months, Mk #2 at 8 months, and Mk #3 at 12 months p.t. with continuous CsA treatment, and Mk #4 at 10 months p.t. with discontinuous CsA treatment. Cell nuclei were counterstained with DAPI. **C** Percentages of CD4^+^ T lymphocytes in human grafts of Mk #1, Mk #2, Mk #3, and Mk#4 shown in (**A** and **B**). **D**, **E** Immunofluorescent analysis of CD8^+^ cytotoxic T (arrow head) lymphocytes in human grafts of Mk #1 at 4 months, Mk #2 at 8 months, and Mk #3 at 12 months p.t. with continuous CsA treatment, and Mk #4 at 10 months p.t. with discontinuous CsA treatment. Cell nuclei were counterstained with DAPI. **F** Percentages of CD8^+^ T lymphocytes in human grafts of Mk #1, Mk #2, Mk #3, and Mk #4 shown in (**D** and **E**). Scale bars: 50 μm in (**A**, **B** and **D**-**E**). Data are represented as the mean ± SEM. *p < 0.05, **p < 0.01, ***p < 0.001, ns (not significant)

Next, we examined CD4^+^ helper T-cells and CD8^+^ cytotoxic T-cells in the grafts. Immunofluorescence and statistical analysis showed that about 20% of cells in the grafts were CD4^+^ helper T-cells at 4 months p.t. (Fig. 3A and C), whereas CD4^+^ T-cells in the grafts at 8 and 12 months p.t. were reduced to about 4% under the continuous administration of CsA (Fig. 3A and C). However, after CsA withdrawal, the percentage of CD4^+^ T-cells in the grafts was increased to more than 30% at 10 months p.t. (Fig. 3B and C).

Similarly, CD8^+^ T-cells occupied ~ 16% of cells in the grafts at 4 months p.t. (Fig. 3D and F). With continuous immunosuppression, CD8^+^ T-cells were decreased to ~ 4% at 8 months p.t., and almost could not be detected at 12 months p.t. in the grafts (Fig. 3D and F). CsA withdrawal resulted in a larger number of resident CD8^+^ T-cells in the grafts (Fig. 3E and F).

Together, these results suggest that continuous immunosuppression could suppress the infiltration of CD4^+^ and CD8^+^ T-cells, while immunosuppressant withdrawal resulted in severe residence of infiltrated helper and cytotoxic T-cells in the grafts.

## Discussion

In this study, we evaluated immune responses in the brains of cynomolgus monkeys with xenografts of human iNPCs under continuous or discontinuous administration of the immunosuppressive drug CsA. According to the histological analyses of immune-responsive cells, we observed strong immune responses shortly after transplantation. In addition, a profound depletion of T-cells and B-cells was further observed after continuous immunosuppression. However, early immunosuppressant withdrawal resulted in severe infiltration of lymphocytes, which suggests that continuous immunosuppression is needed for mitigating immune responses to xenotransplantation.

Cell-based therapy holds great potential for neurodegenerative disease treatment but also faces many obstacles that need to be urgently addressed. Cell transplantation reasonably induces host recognition and response to foreign cells, so the appropriate immune strategy is bound to be established. It has been well documented that autologous cell transplantation and major histocompatibility complex (MHC)-matched primate iPS cell grafts could reduce the host immune response and increase the survival of grafted cells in non-human primate brains (Emborg et al. 2013a; Hallett et al. 2015; Morizane et al. 2017; Tao et al. 2021). However, limited studies have focused on immunosuppressant withdrawal strategies for non-human primates. Previous studies showed that unrejected human PSC-derived neural transplants in parkinsonian rhesus monkey brains were also filled with infiltrating microglia within 1 to 3 months p.t. under daily oral CsA (Emborg et al. 2013b; Kriks et al. 2011), resembling the accumulation and infiltration of massive microglia in the grafts at 4 months p.t. (Fig. 1). After the long-term and continuous duration of immunosuppression, the immune-responsive cells were significantly depleted from 4 months p.t. to 8 months p.t. Until 12 months p.t., microglia nearly declined to normal brain-resident levels (Fig. 1), and lymphocytes were almost to extinction (Fig. 2), consistent with previous observation (Kikuchi et al. 2017) that few or no CD45^+^ lymphocytes infiltrated into human iPS-derived grafts at 12 months p.t. in PD cynomolgus monkeys with long-term Tacrolimus (also a calcineurin inhibitor, CNI) treatment. Additionally, a similar pattern of inflammatory cell infiltration was observed in the co-transplantation of human midbrain dopamine (mDA) cells with autologous regulatory T cells into the mouse striatum, and human mDA cells transplanted alone were rejected without CsA treatment (Park et al. 2023). Although CsA selectively inhibits T-cell proliferation (Halloran et al. 1999), long-term treatment with CsA similarly mitigated the infiltration of B cells (Fig. 2). Collectively, long-term immunosuppression effectively helps the survival of grafts and inhibits the proliferation and accumulation of immune cells.

We also aim to address whether immunosuppressant drugs can be withdrawn after short-term suppression. Our previous work found that the differentiation and maturation of human iNPC-derived neurons were not influenced by immunosuppressant withdrawal (Feng et al. 2022). However, we found that immunosuppressant withdrawal almost caused graft rejection in Mk #5 (data not shown). The graft rejection was possibly caused by the active phagocytic microglia (Fig. 1), which phagocytized the xenografted cells as “pathogen invasion” (Fu et al. 2014). In addition, the infiltration of lymphocytes was extremely robust at 10 months p.t. after immunosuppressant withdrawal (Figs. 2 and 3). One possibility is the recurrence of severe immune responses after immunosuppressant withdrawal. Another is that the immune responses were still strong at 5 months p.t. and unable to be suppressed in the absence of immunosuppressants in the last 5 months. In future studies, more time windows for immunosuppressant withdrawal should be taken into consideration. Thus, our data indicate that the long-term and continuous application of CsA effectively inhibits immune responses in xenotransplantation. Future ideal immunodeficient primates also help tackle the problem of immunosuppression.

Multiple new strategies are in development to shield xenografts in the brain. For example, a transient and systemic blockade of co-stimulation T-cell strategy contributes to efficient human glioblastoma cell engraftment in immunotolerant mice and successful nduction of immunological tolerance towards glial-restricted precursor cells (Lan et al. 2020; Semenkow et al. 2017; Wang et al. 2021). Additionally, modifying the door cells to acquire lower immunogenicity can offer a new opportunity to depress immune responses (Liu et al. 2013; Ozaki et al. 2017). Recent report of co-transplantation with autologous regulatory T cells also provides a potential avenue to achieve better clinical outcomes for cell therapies (Park et al. 2023). Although many efforts have been made to withdraw CNIs from solid organ transplants to avoid long-term side effects, it has been challenging with significantly high risks of graft rejection (Azzi et al. 2013; Mecadon 2021). Instead, the minimization of CNIs may be a safe and long-term beneficial choice (Azzi et al. 2013). Further studies are needed to optimize the minimization protocols, which promise to prevent graft rejection, suppress immune responses, and reduce side effects in cell replacement therapies for neurodegenerative disorders.

## Materials and methods

### Non-human primates

In this study, we recruited five wild-type non-human primates (cynomolgus monkeys, *Macaca fascicularis*) aged 9–13 years old and transplanted human induced neural stem/progenitor cells into the basal forebrain. The care of non-human primates and the experimental procedures were reviewed and approved by the Animal Care and Use Committee of Wincon Theracells Biotechnologies Co., Ltd., in accordance with the Association for Reassessment and Accreditation of Laboratory Animal Care (AAALAC) guidelines. Animals were individually housed in stainless steel cages at the primate facility of Wincon Theracells Biotechnologies Co., Ltd. in Nanning, Guangxi, China, which is fully accredited by AAALAC International. Animals were fed twice daily and supplemented with fresh fruits and miscellaneous enrichments once a day. All animals were maintained on a 12-h light and/or 12-h dark cycle at room temperature at 22–28 °C with a relative humidity of 30%-75% and water supply ad libitum.

### Human iNPC transplantation into the brains of non-human primates

Human iNPCs were generated from human adult peripheral blood mononuclear cells (PB MNCs), which was approved by the Biomedical Research Ethics Committee, Center for Excellence in Molecular Cell Science, Chinese Academy of Sciences, with written informed consent from the donors. The treatment of transcription factors and chemicals to reprogram PB MNCs into NPCs was described in previous studies (Zhang et al. 2019, 2021). Human iNPCs were labeled with GFP, and neural spheres at passage 15 were dissociated into single cells using Accutase and suspended in neural differentiation medium, serving as donor cells to be bilaterally delivered into the basal forebrain of cynomolgus monkeys. The precise injection site was identified by MRI scanning. A total of 8 million cells were injected into four sites of the forebrain using a Hamilton syringe (gauge 22 s) by MRI-guided stereotaxic surgery combined with a convection-enhanced delivery system, as described previously (Feng et al. 2022; Yue et al. 2021). Non-human primates were anesthetized with intramuscular atropine (20 mg/kg), ketamine (10 mg/kg), and sodium pentobarbital (20 mg/kg), and their heads were fixed by a stereotaxic instrument during surgery. The syringe with human iNPC suspension was pushed from a small hole into the target site at a rate of 1 mm/min and held in place for 10 min. Then, the suspension was delivered to the target site at a rate of 1 μl/min. After holding in place for 20 min, the syringe was drawn back at a rate of 1 mm/min. The wound was stitched, and the non-human primates were taken good care of by veterinarians.

### Immunosuppression strategy

To avoid the immunological rejection of xenografts, non-human primates were administered with the immunosuppressant CsA. Two days before surgery, non-human primates began to take cyclosporin A at a dose of 30 mg/kg. After surgery, the dose of CsA was decreased by 5 mg/kg per week until it was reduced to 15 mg/kg at the fourth week. For 3 non-human primates (Mk #1, #2, and #3), CsA administration was maintained at 15 mg/kg until euthanasia. Unlike the above monkeys, Mk #4 and Mk #5 only received CsA administration in the first 5 months, and immunosuppressant withdrawal was performed from the sixth month.

### Immunostaining

Mk #1, #2, and #3 were perfused with saline under deep anesthesia with sodium pentobarbital (30 mg/kg intravenously), and then the brains were dissected out. Whole brains were coronally cut into 4 mm thick blocks, which were then fixed with 4% paraformaldehyde (PFA) for 3 days at 4 °C. After three washes with PBS, the blocks were sequentially immersed in 15% and then 30% sucrose solutions at 4 °C for dehydration. Brain blocks were further cryo-sectioned into brain slides of 40 μm thickness using a microtome (Leica SM2000R) and stored in ethylene glycol solutions at -20 °C. Mk #4 and #5 were deeply anesthetized, and brains were directly collected. The brain blocks harboring GFP^+^ grafts were sliced coronally into 350 μm thick sections with a vibratome (Leica VT1000S) for electrophysiological recording in a previous study (Feng et al. 2022). The recorded slices were further cryo-sectioned into 15 μm thick sections for immunostaining.

For immunostaining, single staining of IBA1, CD68 or HLA-DR was performed according to the normal protocol, while co-staining of lymphocyte markers was performed using a Four-color fluorescence kit (Recordbio Biological Technology, Shanghai, China) based on tyramide signal amplification (TSA) technology according to the manufacturer’s instructions. The primary antibodies used were as follows: polyclonal goat anti-IBA1 (Abcam, ab5076, 1:500), monoclonal mouse anti-CD68 (Thermo Fisher Scientific, MA5-13,324, 1:500), monoclonal mouse anti-HLA-DR (Abcam, ab136320, 1:500), polyclonal rabbit anti-CD3 (Abcam, ab5690, 1:2000), polyclonal rabbit anti-CD4 (Abcam, ab288724, 1:2000), monoclonal rabbit anti-CD8 (Thermo Fisher, MA5-14,548, 1:2000), monoclonal rabbit anti-CD19 (Abcam, ab134114, 1:2000), polyclonal rabbit anti-GFP (Thermo Fisher Scientific, A-11122, 1:200), polyclonal rabbit-anti-NF-H (Proteintech, 18,934–1-AP, 1:800), and monoclonal rabbit-anti-SYNAPTOPHYSIN (Abcam, ab32127, 1:500). To reduce tissue autofluorescence, brain slides were dipped briefly in distilled water and then treated with 5 mM CuSO4 in 50 mM ammonium acetate buffer (pH 5.0) for 30 min. DAPI was used to counterstain nuclei. Confocal images were collected with confocal laser scanning microscopes (Leica TCS SP8 STED and Olympus FV3000). Each image was scanned to a thickness of 10 μm and then processed. For quantification, at least 5 fields were randomly chosen for each primate.

### Statistical analysis

For cell counting of IBA1^+^, CD19^+^, CD3^+^, CD4^+^, and CD8^+^ cells, at least three fields were randomly chosen for each monkey and quantified manually. The positive areas of CD68 and HLA-DR were measured by StrataQuest software version 6.0.1.145 (TissueGnostics, Vienna, Austria). The quantification was performed on at least three fields from each primate.

All statistical analyses were performed in GraphPad Prism software (GraphPad 9.0). Data are presented as the mean ± SEM. Student’s *t* test (two tailed) was performed for statistical analysis between every two groups. Statistical significance was set at *p < 0.05, **p < 0.01, ***p < 0.001, and ****p < 0.0001.

### Supplementary Information

**Additional file 1:Figure S1.** The identification of transplanted human iNPCs-derived neurons and the expression of SYNAPTOPHYSIN in human cell grafts of monkey brains under discontinuous treatment of CsA. **Figure S2.** The human grafts in monkey brains under continuous and discontinuous treatment of CsA. **Figure S3.** The infiltration of HLA-DR+ microglia/macrophages into human grafts.

## Data Availability

Not applicable.

## Abbreviations

iNPCs: induced neural stem/progenitor cells
p.t.: Post transplantation
AD: Alzheimer’s disease
PD: Parkinson’s disease
ALS: Amyotrophic lateral sclerosis
iPS cells: induced pluripotent stem cells
CsA: Cyclosporin A
IL-2: Interleukin 2
PB MNCs: Peripheral blood mononuclear cells
HLA-DR: Human leukocyte antigen-D related
MHC: Major histocompatibility complex
CNI: Calcineurin inhibitor
mDA: Midbrain dopamine
Mk: Monkey
