# Supplementary material for: Continuous immunosuppression is required for suppressing immune responses to xenografts in non-human primate brains
Source: Cell Regen. 2024 Apr 7;13:8. doi: 10.1186/s13619-024-00191-0 (PMC10999398; doi:10.1186/s13619-024-00191-0)
Supplement: Supplementary file 1 — Additional file 1:Figure S1. The identification of transplanted human iNPCs-derived neurons and the expression of SYNAPTOPHYSIN in human cell grafts of monkey brains under discontinuous treatment of CsA. Figure S2. The human grafts in monkey brains under continuous and discontinuous treatment of CsA. Figure S3. The infiltration of HLA-DR+ microglia/macrophages into human grafts. [file 13619_2024_191_MOESM1_ESM.pdf]

Figure S1

The identification of transplanted human iNPCs-derived neurons and the expression of SYNAPTOPHYSIN in human cell grafts of monkey brains under discontinuous treatment of CsA.

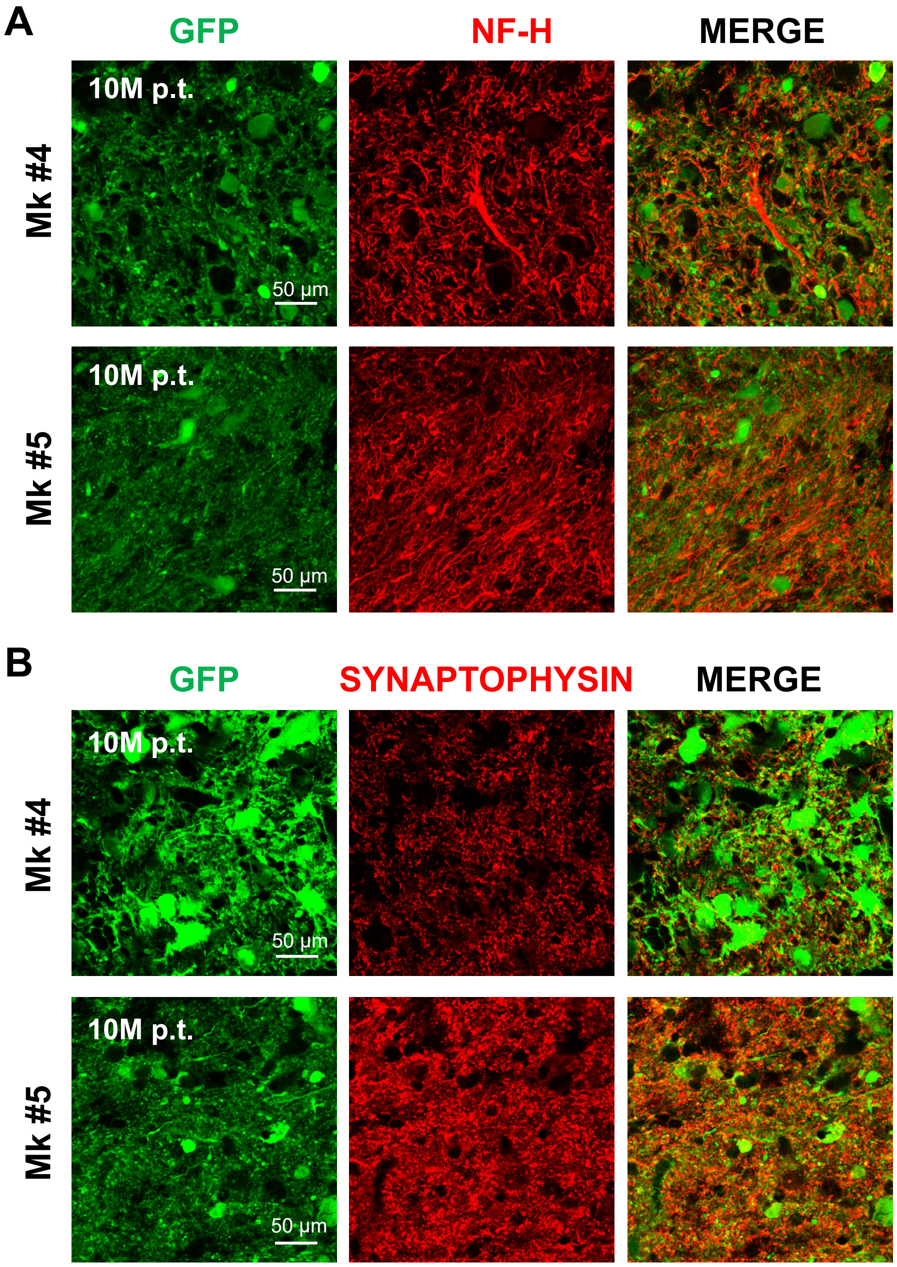

Figure legends:

A. Immunofluorescent detection of NF-H⁺ neurons in human grafts of Mk #4 and Mk #5 at 10 months p.t. under discontinuous CsA treatment.

B. Immunofluorescent detection of presynaptic protein SYNAPTOPHYSIN in human grafts of Mk #4 and Mk #5 at 10 months p.t. under discontinuous CsA treatment.

Scale bars: 50 μm in A and B.

Figure S2│

The human grafts in monkey brains under continuous and discontinuous treatment of CsA.

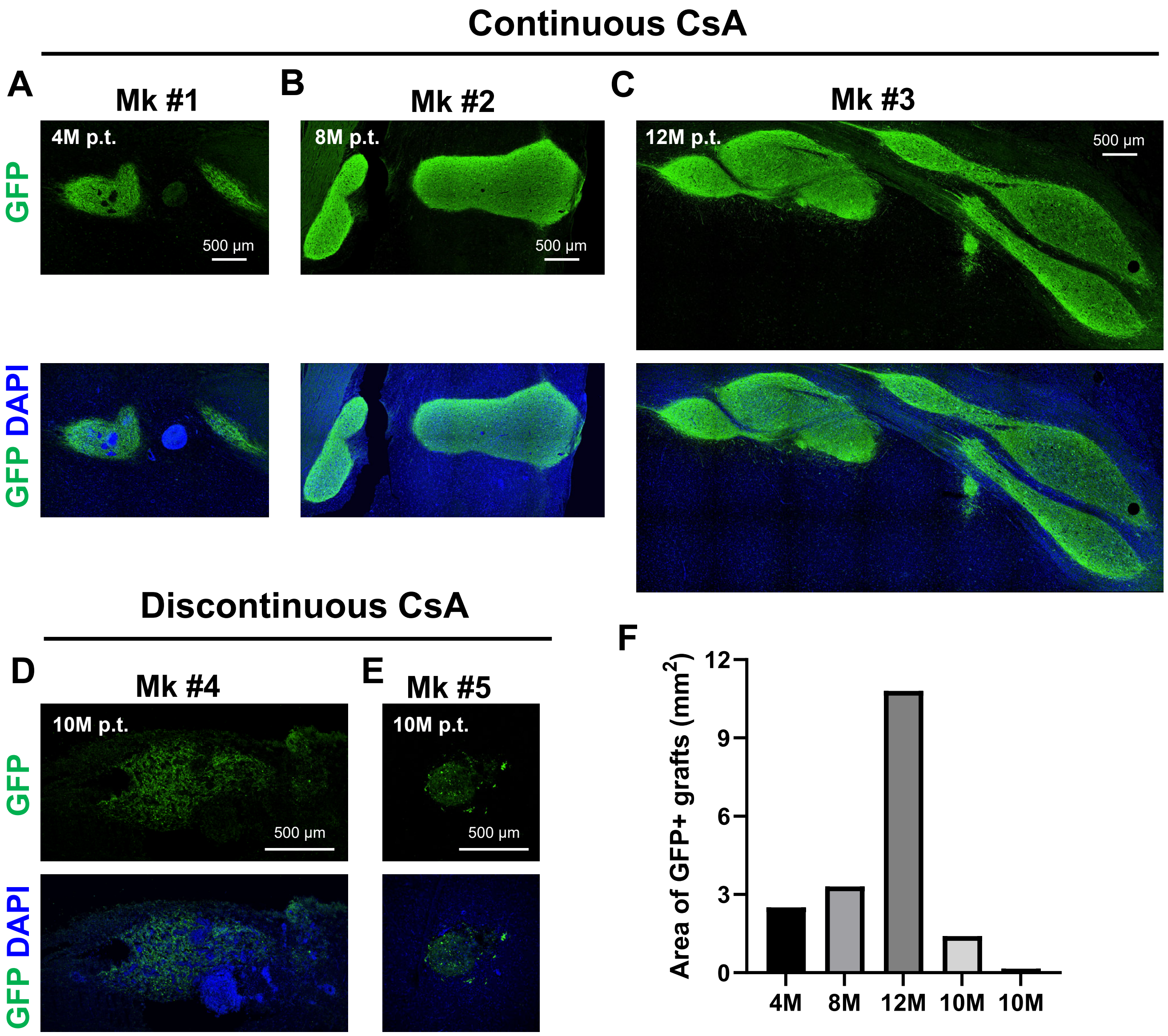

Figure legends:

A-C. The lower magnifying power images of whole GFP⁺ grafts in the hemispheres of transplanted Mk #1 at 4 months, Mk #2 at 8 months, and Mk #3 at 12 months p.t. under continuous CsA treatment. Cell nuclei were counterstained with DAPI.

D-E. The lower magnifying power images of whole GFP⁺ grafts in the hemispheres of transplanted Mk #4 and Mk #5 at 10 months p.t. under discontinuous CsA treatment. Cell nuclei were counterstained with DAPI.

F. The estimated area of GFP+ grafts showed in A-E.

Scale bars: 500 μm in A-E.

Figure S3

The infiltration of HLA-DR+ microglia/macrophages into human grafts.

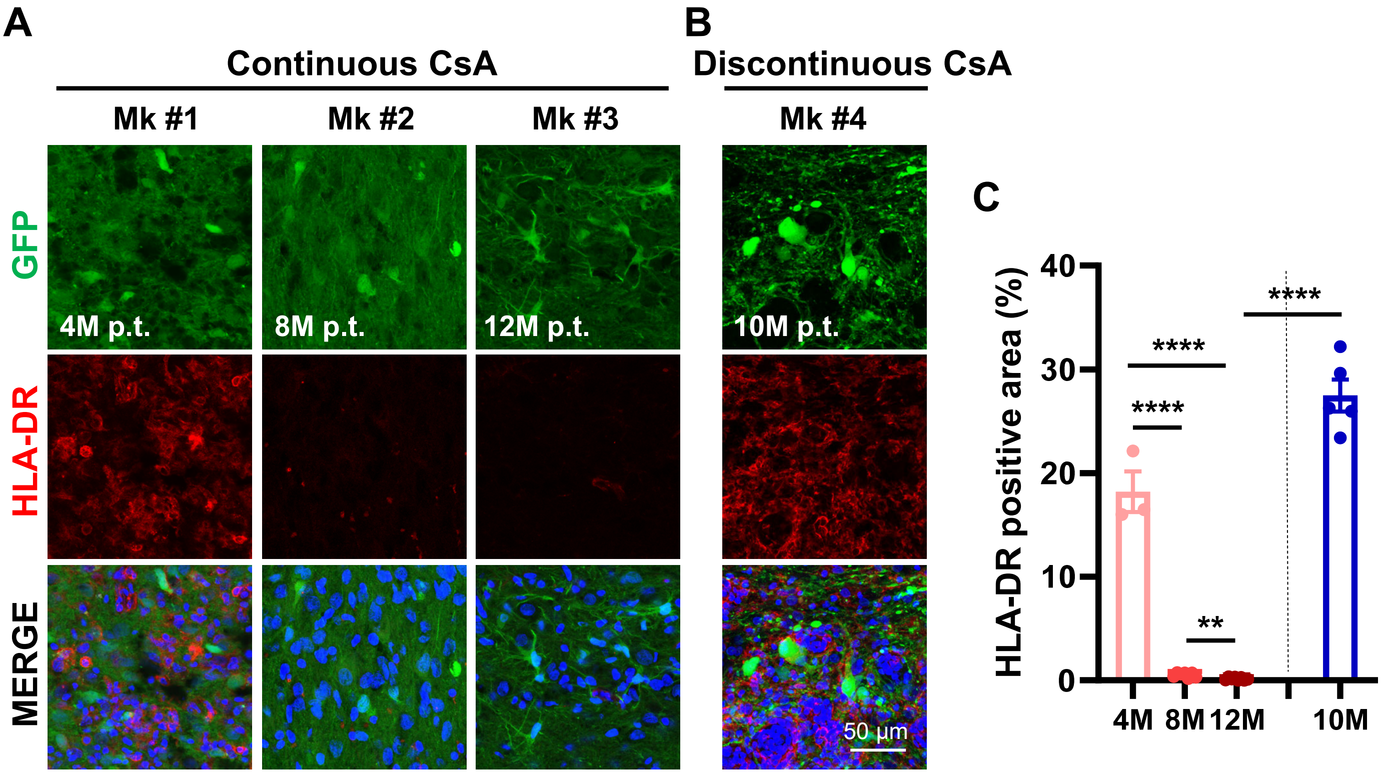

Figure legends:

A-B. Immunofluorescent analysis of HLA-DR⁺ microglia/macrophages in human grafts of Mk #1 at 4 months, Mk #2 at 8 months, and Mk #3 at 12 months p.t. under continuous CsA treatment, and Mk #4 at 10 months p.t. under discontinuous CsA treatment. Cell nuclei were counterstained with DAPI.

C. Quantification of HLA-DR positive area percentage per field shown in A-B.

Scale bars: 50 μm in A-B and D-E. Data are represented as the mean ± SEM. **p < 0.01, ****p < 0.0001.
